# Characterization of the chloroplast genome of *Lagerstroemia villosa* Wall. ex Kurz. (*Lagerstroemia*, Lythraceae)

**DOI:** 10.1080/23802359.2020.1844095

**Published:** 2021-01-06

**Authors:** Jie Wang, Zhi-Qiang Wu, Li Ma, Cui-Hua Gu

**Affiliations:** a School of Landscape and Architecture, Zhejiang A & F University, Hangzhou, China; b Zhejiang Provincial Key Laboratory of Germplasm Innovation and Utilization for Garden Plants, Zhejiang A & F University, Hangzhou, China; c Key Laboratory of National Forestry and Grassland Administration on Germplasm Innovation and Utilization for Southern Garden Plants, Zhejiang A & F University, Hangzhou, China; d Shenzhen Branch, Guangdong Laboratory for Lingnan Modern Agriculture, Genome Analysis Laboratory of the Ministry of Agriculture, Agricultural Genomics Institute at Shenzhen, Chinese Academy of Agricultural Sciences, Shenzhen, China

**Keywords:** *Lagerstroemia villosa*, chloroplast genome, phylogeney

## Abstract

*Lagerstroemia villosa* is a kind of ornamental tree with surprising potential for applying in the landscape. We characterized the complete chloroplast genome of this scarce species and analyzed its phylogeny within Lythraceae. The result showed that the genome possessed a typical quadripartite structure, in more detail, a lager single-copy region (LSC, 88,702bp), a small single-copy region (SSC, 18,255bp), and a pair of inverted repeat regions (IRa and IRb, 26,906 bp). 78 protein-coding genes, four ribosomal RNA (rRNA) genes, and 30 transfer RNA (tRNA) genes were detected. Phylogenetic analysis based on maximum likelihood (ML) supported the closest relationship between *L. villosa* and *Lagerstroemia limii* plus *Lagerstroemia subcostata*.

*Lagerstroemia villosa* Wall. ex Kurz. (Lagerstroemia, Lythraceae) is a kind of tall tree and can reach 10–15 meters in height, whose branchlets, both surfaces of leaves and inflorescence were covered with fine, white pubescent. It is usually distributed in mixed forests at an altitude of 700–1000 meters in nature, and mainly concentrated in Southeast Asia, i.e. Yunnan Province of China, and part areas of Vietnam, Myanmar, and Thailand as well (Qin and Shirley 2007). Similar to other *Lagerstroemia* species, *L. villosa* possesses ornamental value for its adorable, elegant light purple flowers blooming during the summer, which accounts for its potential to be widely used in the landscape. Unfortunately, the phenomenon that its population is decreasing due to the habitat destruction has not attracted enough attention (De Wilde and Duyfjes 2016). Accordingly, we characterized the complete chloroplast genome of this delicate species, with a hope to promote its wild protection and make its large-scale application possible.

Fresh leaves of *L. villosa* were sampled in the nursery of Zhejiang A & F University, Hangzhou, Zhejiang province, China (30°13′48″N, 119°43′12″E), which were stored as the specimen (Code: ZAFU1913141) in the Herbarium of Zhejiang A & F University. The total genomic DNA was extracted following the method proposed by Doyle (1987) and Yang et al. (2014). After establishing a sequencing library with an average insert size of 350 bp based on total DNA, 6.78 GB raw data with 150 bp paired-end reads were preliminarily obtained via Illumina HiSeq platform (Shenzhen, China), and subsequently filtered utilizing Trimmomatic v0.3 (Bolger et al. 2014). The *de novo* assembly process was accomplished by CLC v9.11 (Nicolas et al. 2017). The contigs alignment was performed under the BLAST algorithm (Johnson et al. 2008), taking *Lagerstroemia indica* plastid genome as reference (Xu et al. 2017). Finally, we annotated the genome using DOGMA v1.2 (Wyman 2004) and submitted it to GenBank (Accession number: MK881633). Additionally, MISA-web v2.1 (Beier et al. 2017) was applied to identify simple sequence repeats sequences (SSR).

The chloroplast genome size reached 160,769 bp with quadripartite structure, namely, a lager single-copy region (LSC) of 88,702 bp and a small single-copy region (SSC) of 18,255 bp, separated by two inverted repeat regions (IRa and IRb) of 26,906 bp. The overall GC content was 36.97%, while 34.69%, 30.78%, and 42.83% were respectively corresponding to that of LSC, SSC, and IR. Among identified 325 SSRs, 212 were mono-nucleotide (65.23%), 45 were di- (13.85%), 61 were tri- (18.77%), 6 were tetra- (1.85%), and 1 (0.31%) were penta-, respectively. A total of 112 unique genes comprised of 78 protein-coding genes, four ribosomal RNA (rRNA) genes, and 30 transfer RNA (tRNA) genes, among which 14 genes contained one intron while three (*rps12*, *ycf3*, and *clpP*) contained two.

Additional 14 related species of Lythraceae were selected from NCBI for investigating the phylogeny of *L. villosa*, together with another two species of Onagraceae as outgroups. MAFFT v7 (Katoh et al. 2019) was utilized to implement chloroplast genome alignment. The best fit model (GTR + F + R2) was confirmed through ModelFinder (Kalyaanamoorthy et al. 2017), and then Maximum likelihood (ML) analysis was carried out via IQ-TREE module in PhyloSuite (Zhang et al. 2020). It showed that *L. villosa* was nested within the *Lagerstroemia* spp. calde and had the closest relationship with *Lagerstroemia limii* and *Lagerstroemia subcostata* (Figure 1).

**Figure 1.**
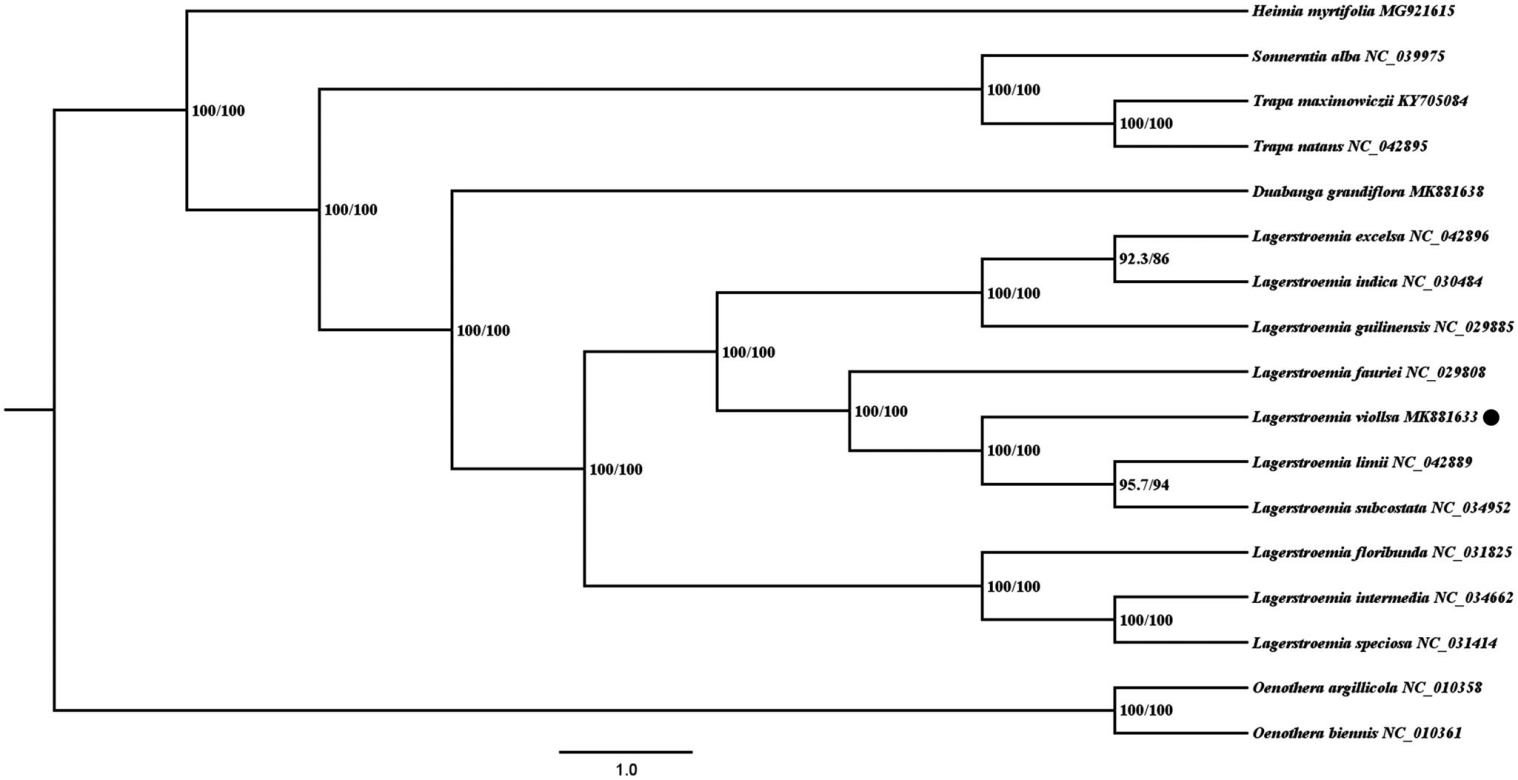
ML analysis based on 15 Lythraceae species with two Onagraceae species as outgroups. The numbers above branches indicated the value of Sh-aLRT and Boostrap, respectively.

## Data Availability

The data that support the findings of this study are openly available in GenBank at https://www.ncbi.nlm.nih.gov/genbank/, reference number: MK881633.
